# Salmagundi

**Published:** 1887-09

**Authors:** 


### SALMAGUNDI.

EDITED BY E. G. BETTY, D.D.S.

RESECTION of the pylorus has been performed by Billroth fifteen times for carcinoma, with success in seven cases.

A TOOTH immersed in a solution of the tincture of iron in eight parts of water has its enamel entirely destroyed in one hour. —*Exchange.*

MORE deaths occur from lockjaw on Long Island, it is stated, than upon any other corresponding area of territory in this country.—*Clipping.*

CAN DOCTORS PRACTICE DENTISTRY?—A case involving this point recently came up in New York, and was decided in the affirmative. A Dr. Bradford was charged with illegally practicing dentistry. He showed that he was a legal physician and the complaint was dismissed.—*Med. and Surg. Rep.*

COMPLAINT has recently been made of the abuse of the Clinic in dental colleges, the college authorities giving services practically for nothing to people abundantly able to pay the regular office fees. Such people should be refused point blank and thus made to seek professional advice and service at the hands of a regular practioner, thereby preventing the abuse of the Clinic which is for the mutual good of the student and the impecunious. The alarming extent to which this abuse has been carried in medical clinics is evidenced by the following clipping from the *Medical and Surgical Reporter*:

A recent estimate shows that about one-fourth of the population of New York, Boston, and London receive free treatment at the medical clinics; in Philadelphia, one-fifth, and in Liverpool over one-half the population.

